# Antibiotic Treatment vs. Non-Antibiotic Treatment in Bovine Clinical Mastitis During Lactation with Mild and Moderate Severity

**DOI:** 10.3390/antibiotics14070702

**Published:** 2025-07-12

**Authors:** Franziska Nankemann, Stefanie Leimbach, Julia Nitz, Anne Tellen, Nicole Wente, Yanchao Zhang, Doris Klocke, Isabel Krebs, Stephanie Müller, Sabrina Teich, Jensine Wilm, Pauline Katthöfer, Jan Kortstegge, Volker Krömker

**Affiliations:** 1 Department of Microbiology, Faculty of Mechanical and Bioprocess Engineering, University of Applied Sciences and Arts, 30453 Hannover, Germany; franziska.nankemann@hs-hannover.de (F.N.); stefanie.leimbach@hs-hannover.de (S.L.); julia.nitz@hs-hannover.de (J.N.); anne.tellen@hs-hannover.de (A.T.); nicole.wente@hs-hannover.de (N.W.); yanchao.zhang@hs-hannover.de (Y.Z.); doris.klocke@hs-hannover.de (D.K.); isabel.krebs@web.de (I.K.); stephanie.mueller@tiho-hannover.de (S.M.); sabrina.teich@gmail.com (S.T.); pauline.katthoefer@tiho-hannover.de (P.K.); jan.kortstegge@tiho-hannover.de (J.K.); 2 Department of Veterinary and Animal Sciences, University of Copenhagen, 1870 Frederiksberg C, Denmark; jensine.wilm@sund.ku.dk

**Keywords:** mastitis, antibiotic therapy, spontaneous cure, bacteriological cure

## Abstract

Background/Objectives: This review aimed to compare the efficacy of antibiotic treatment vs. non-antibiotic treatment in mild and moderate clinical mastitis in lactating dairy cows, categorized by the causative pathogen. Methods: The initial systematic review plan, which resulted in only four relevant articles, was altered due to limited available studies and significant heterogeneity among them. Consequently, five additional articles, closely meeting our criteria with minor differences, were included to ensure comprehensive analysis, resulting in nine included articles. Due to these pragmatic constraints, this review represents a hybrid between a systematic and a narrative review. The outcome of interest was the bacteriological cure (BC). Results: The findings revealed that antibiotic treatment resulted in improved BC rates for cases caused by Streptococci. For cases caused by *Escherichia (E.) coli*, antibiotic therapy showed no significant improvement in BC rates compared to non-antibiotic treatment, suggesting that antibiotics may be often unnecessary for these cases due to self-limiting tendencies. However, severe *E. coli* mastitis warrants systemic antibiotic treatment due to potentially life-threatening complications. *Klebsiella* spp. mastitis showed better cure rates with antibiotic therapy. Conclusions: This study underscores the importance of regular pathogen diagnostics to guide appropriate treatment, advocating for the use of on-farm rapid tests to reduce unnecessary antibiotic use while ensuring effective treatment outcomes.

## 1. Introduction

To protect human and animal health by preventing the development of antibiotic resistance, it is crucial to promote the prudent use of antibiotics. In dairy cattle, bovine mastitis is accountable for the majority of antibiotics (>70%) used [1]. During lactation, it is recommended to treat clinical mastitis (CM) in a targeted manner. In contrast, the treatment of subclinical mastitis (SCM) during lactation is recommended at dry-off rather than in lactation. For this reason, the treatment of CM has the potential to reduce the overall antibiotic consumption in cattle production in lactation [2].

Targeted antibiotic therapy of only those mastitis cases that benefit from antibiotic therapy must be the goal to consider animal welfare and farm profitability [2]. Antibiotic substances can either prevent bacteria from developing in the target tissue or eliminate them altogether. Accordingly, the efficacy of antibiotic therapy in bovine mastitis must be measured primarily by the bacteriological cure (BC) rate. This varies considerably and is influenced by various factors, including the severity of clinical signs, pathogen factors, and animal factors [3]. A recent meta-analysis demonstrated that published selective treatment protocols for CM, which include the factors just mentioned, are without adverse effects on bacteriological and clinical cure, somatic cell count (SCC), milk yield, and the incidence of recurrence or culling [4]. The advancement of targeted treatment protocols published to date relies on a comprehensive understanding of all factors that impact BC. By taking these factors into account when making therapeutic decisions, antibiotic substances can be used more effectively, allowing for more prudent use of antibiotics.

In practice, the categorization of clinical symptoms by severity (mild, moderate, or severe) is used as a common tool for categorization and decision-making, with a mild case representing a change in the visual appearance of the milk, a moderate case including signs of inflammation in the respective udder quarter, and a severe case defined by additional systemic signs of disease [5]. Cases with severe signs are considered to require immediate systemic antibiotic treatment to reduce the risk of bacteremia, while mild to moderate cases, presenting about 85% of clinical cases [6], leave more time for diagnosis before therapy [7,8].

Antibiotic therapy for mastitis is based on the assumption that intramammary infections with microorganisms (bacteria, fungi, and algae) are prerequisites for mastitis. However, mastitis pathogens cannot be detected in all mastitis cases, and not all mastitis-causing microorganisms are susceptible to antimicrobial therapy. In addition to the varying susceptibility of mastitis pathogens to antimicrobial agents, the BC initiated by antibiotic therapy may or may not significantly enhance the underlying spontaneous cure rate. Spontaneous cure rates are thought to range from low (*Staphylococcus (S.) aureus*) to high (*Escherichia (E.) coli*) [3]. This is why antibiotic treatment is generally not recommended in mild and moderate cases of mastitis caused by Gram-negative bacteria such as *E. coli*. The same applies to cases in which no microbiological growth can be detected. In those cases of CM where antibiotic treatment does not improve the chances of BC, due to already high spontaneous cure rates or resistance against antibiotic substances, the value of supportive treatment like non-steroidal anti-inflammatory drugs (NSAIDs) has been emphasized for many years now [3,9,10]. Although no non-antibiotic treatment could be shown to affect overall cure rates within a previous systematic review carried out in 2017 [11], various studies have been performed since then and demonstrated the benefit of some supportive treatments on BC among other outcome measures [12,13].

Several papers have shown the importance of animal factors in BC rates [14,15]. Animals that are chronically udder-diseased because they had an SCC of ≥700,000 cells/mL of milk in three consecutive milk controls or had two or more CM cases in the current lactation are considered unworthy of local antibiotic treatment. It has been proven that BC cannot be increased by antibiotic treatment in these animals. However, these animal factors are not considered often enough in therapy studies.

A review comparatively describing the BC rate of relevant pathogen groups after antibiotic therapy and after non-antibiotic or placebo therapy in the context of randomized clinical trials has been missing to date. Therefore, this study aims to include randomized controlled trials (RCTs) comparing intervention groups with antibiotic treatments to non-antibiotic treatments, as well as negative controls, in cases of bovine mild and moderate CM stratified by the causative pathogen group. This investigation could serve as a basis for evaluating the benefits of antibiotic treatment and formulating recommendations to help decision-makers apply antibiotics only to cases that may benefit from treatment.

### Objective

As part of the European Network for Optimization of Veterinary Antimicrobial Treatment (ENOVAT), a group of researchers and key opinion leaders from various European countries (https://enovat.eu/ accessed on 1 January 2022) is working to establish basic guidelines for antibiotic mastitis treatment that can be used in the development of country-specific guidelines. The working group includes pharmacologists, epidemiologists, microbiologists, and cliniciansn and is gender-balanced. The guideline development process is preceded by identifying research questions that are considered essential for guideline formulation. During the joint discussion, the following research question for a review was identified: How does antibiotic treatment (local and/or systemic) perform relative to non-antibiotic treatment or no treatment, measured by BC, following cases of CM in lactating dairy cows of mild and moderate severity stratified by the causative pathogen? The objective of this study is to answer this question and provide baseline conclusions that can serve as the basis for developing clinical recommendations on the targeted use of antibiotic therapy.

This question can be further described by the four PICO elements:

Population  Lactating cows with mild/moderate mastitis

Intervention  Antibiotic therapy (local and/or systemic)

Comparator  Non-antibiotic treatment and negative controls

Outcome  Bacteriological cure

The main objective is thereby not to determine the absolute cure rate, but rather the relative difference in BC for different pathogen groups between the antibiotic-treated groups and the non-antibiotic treatment or no-treatment groups.

## 2. Results

### 2.1. Study Selection

**Figure 1:**
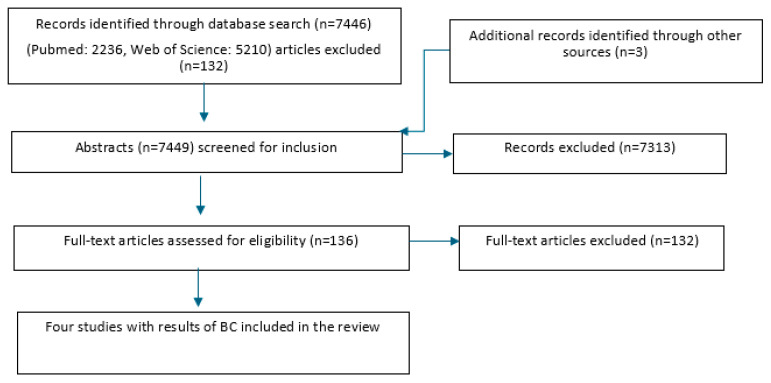
Study flowchart for the search and subsequent selection of eligible articles.

In total, the search on PubMed yielded 2236 possible articles, and Web of Science an additional 5210 (Figure 1). Three more articles were found from other sources. Thus, the search strategy yielded a total of 7449 possible articles. After the first step of the selection process (abstract and title screening), a total of 136 articles remained to proceed to the second step. After full-text screening by the junior and senior groups, a total of four articles remained. Articles were excluded for the following reasons: language, no control group, experimentally induced infections, no clinical trial, severity unclear, article not procurable, determination of cure below 14 days after treatment, review without new sources not yet covered by search in Pubmed/WoS, no results for BC for the control group, no cure rates given for different pathogens, results for clinical and subclinical cases reported together, and different study purposes.

### 2.2. Description of the Included Studies

A description of important characteristics of the studies can be seen in Table 1. All studies analyzed in this review included only mild and moderate mastitis cases, according to the eligibility criteria for this review. However, each study had its own set of additional criteria when selecting suitable animals to participate. Guterbock et al. [16] included only animals that had not been vaccinated against mastitis, were milked twice daily, and had a bulk tank SCC below 200,000/mL milk. In the study by Schukken et al. [17], animals were not allowed to have received an antibiotic treatment in the 14 days prior. In addition, this study only included animals of which one quarter was affected and no animals that had lesions on the teats. Guterbock et al. [16] also included only animals with one affected quarter. In the other studies, multiple quarters were allowed to be affected. In the study by Fuenzalida and Ruegg [18], multiple quarters were included only if all were Gram-negative and the on-farm test did not vary between quarters. Animals that had other diseases requiring treatment were also excluded from the study by Roberson et al. [19]. Only Gram-negative cases were included in studies by Schukken et al. [17] and Fuenzalida and Ruegg [18]. In all studies, severe mastitis cases, defined as cases associated with systemic disease symptoms, were excluded. Further exclusion criteria were valuable animals and cows expected to be culled before reaching day 21 (last sampling day). Cases caused by Gram-positive pathogens or cases with a negative culture result of the on-farm test were not included in the studies by Schukken et al. [17] and Fuenzalida and Ruegg [18].

The studies all followed a split-herd design, where cases of CM were divided into those treated with antibiotics and those receiving a non-antibiotic treatment and/or no treatment. The severity of CM cases was primarily determined using the IDF’s classification system, with mild cases being characterized by visual changes in milk and moderate cases including inflammation in the udder [5]. However, in Roberson et al. [19], the severity of CM cases was evaluated using a unique protocol that considered eight different parameters, such as rectal temperature, heart rate, and respiratory rate. Mild cases were only diagnosed if none of these parameters showed abnormal values, and moderate cases were defined as having one abnormal parameter. In Schukken et al. [17], a mild case could have one systemic sign, which was a unique aspect of that particular study.

The diagnosis of CM was determined by the farm personnel, including milking technicians, in all studies. The majority of the studies used culture diagnostics to identify mastitis pathogens. Schukken et al. [17] used bacteriological culturing and, in some cases, molecular typing. At case enrollment, the identification of Gram-negative pathogens was performed using on-farm culture in the studies by Schukken et al. [17] and Fuenzalida and Ruegg [18], and subsequently confirmed in a laboratory. The outcome of interest, BC, was measured at different time points throughout the studies, but there was always at least one time point that was a minimum of 14 days after treatment onset, thus conforming to our specifications. All studies measured BC at two time points (Guterbock et al. [16] at day 5/day 6 (milking 9/11) and day 21, Roberson et al. [19] at day 7 and day 36, Schukken et al. [17] at day 7 and day 14, and Fuenzalida and Ruegg [18] at day 14 and day 21) (Table 1).

Various antibiotic agents were used, but all of these were used intramammarily (Table 2). In one study, they compared two different antibiotics (amoxicillin and cephapirin) with oxytocin in the control group [16]. Roberson et al. [19] used only amoxicillin, but the dosage and duration were the same as in Guterbock et al. [16]. Two studies explicitly examined only Gram-negative cases, both using ceftiofur hydrochloride with varying durations but the same dosage [17,18]. All studies showed no evidence of resistance to the antibiotics used.

In total, there were two studies that compared the efficacy of antibiotic treatment and a negative control [17,18]. The non-antibiotic treatment used was oxytocin [16]. In Roberson et al. [19], antibiotic treatment was compared, on the one hand, with no treatment, and on the other hand, with frequent milk-out in combination with 20 international units (IUs) of oxytocin.

### 2.3. Risk-of-Bias Assessment

The results of the bias analysis are shown in Table 3. The total risk of bias was determined by the worst result in each domain. Out of the four articles, three received a low risk of bias, while one received an increased risk of bias.

The study by Guterbock et al. [16] had an elevated risk of bias due to the randomization process (domain 1). In this study, not all animals were included, as valuable animals for the farmer and animals that were to be culled before the end of the 21 days interval were excluded from the study. Furthermore, milk yield was significantly different between treatment groups, so the groups were not comparable.

The measurement of the outcome (domain 4) showed an elevated risk of bias in the study by Guterbock et al. [16] as well. Control milk samples for the group receiving amoxicillin were taken at milking 9 and for the Cephapirin group at milking 11. Another control sample was taken on day 21 in all treatment groups. Sampling at different times was evaluated with some concerns because different time points can lead to different effects due to possible impacts of antibiotic residues or, conversely, to an increasing risk of new infections as more time passes since the treatment.

### 2.4. Descriptive Outcomes of Bacteriological Cure for Specific Pathogens

The results of BC stratified by pathogen group are shown in Table 4. In all studies, BC was defined as the absence of the pathogen found in the pretreatment sample in all post-treatment samples. Sufficient data could only be found for streptococci and coliform microorganisms to compare the BC rates.

According to the study by Roberson et al. [19], higher cure rates for intramammary infections with streptococci were recorded for the antibiotic treatment group compared to the other treatment and no treatment. Amoxicillin treatment resulted in a 60% cure rate for BC on day 7, compared to only 8% and 29% for frequent milk-out and no treatment, respectively. By day 36, the antibiotic treatment group had a 75% BC cure rate, while the comparator groups had only 22% and 29% cure rates. In the study by Guterbock et al. [16], there was no difference in BC rates for streptococci in cases treated with amoxicillin or oxytocin (46.2% vs. 47.6%). However, Cephapirin treatment showed a tendency toward higher cure rates (73.3%).

In contrast, there were different results for the cure rates of cases due to coliforms. Both the studies by Fuenzalida and Ruegg [18] and Roberson et al. [19] found no differences in BC between the treatment groups in cases caused by *E. coli*. One study that reported results for coliforms also found no difference in BC between the treatment groups [16]. However, Schukken et al. [17] found significantly higher cure rates in the antibiotic treatment group (89%) compared to the untreated group (53%) for cases caused by *E. coli*. Antibiotic treatment was more effective regarding BC in CM caused by *Klebsiella* spp. Both antibiotic treatments in the study by Fuenzalida and Ruegg [18] (for cases caused by *Klebsiella pneumoniae)* and the administration of ceftiofur in Schukken et al. [17] (*Klebsiella* spp.) resulted in higher BC than the untreated control. In the Roberson et al. [19] study, no satisfactory BC results were found for *Klebsiella* spp. mastitis cases in any treatment group.

### 2.5. Deviation from the PRISMA Path: Exploring Relevant Research Beyond Initial Systematic Review Constraints

We had aimed to carry out a systematic review and had formally followed the process. In the end, however, we ended up with only four papers, which were also highly heterogeneous and therefore did not allow any valid conclusions to be drawn. During the full-text screening, we found other papers that are modern, performed well, and provide valid statements. Moreover, they differed only marginally from our criteria. For this reason, instead of arriving at no results, we decided to leave the defined path of PRISMA-based systematic reviews and extend the analysis by five articles [20,21,22,23,24]. Due to these pragmatic constraints, it should be clarified that this review represents a mixture of systematic and narrative approaches.

In the study by Keller and Sundrum (2018) [20], the BC rate at the pathogen level could only be calculated on day 7 due to low sample sizes. Their study was performed on four German dairy herds, one organic and three conventional. They included 180 dairy cows with 60 in each treatment group. They compared antibiotic treatment with homeopathic treatment (21 different remedies) or placebo treatment (globuli sacchari). The antibiotics used were chosen according to the veterinarian, but all substances were regularly used in Germany. In the other four studies [21,22,23,24], severe cases were also included, and the results were not always reported separately for the different severity levels. The most important characteristics of these studies are shown in Table 5.

Table 5, showing the BC results of the original four articles, was expanded with the results of the five additional articles, and the results can be seen in Table 6. The results of the additional articles for streptococci are consistent with the results of the original articles described above. In the study by Morin et al. [23], a BC rate of almost 71% was achieved in the antibiotic treatment group, in contrast to approximately 28% in the control group. In the study by Keller and Sundrum [20], antibiotic treatment resulted in significantly higher BC rates compared to placebo and homeopathic treatment regarding cases caused by *S. uberis* (64.7% vs. 18.7% and 8.3%) and *S. dysgalactiae* (85.7% vs. 66.7% and 0.0%). There were no differences between the antibiotic treatment group and the control group in the study by Klostermann et al. [22] in cases caused by S. dysgalactiae (both 40%). However, antibiotic treatment performed better in cases caused by *S. uberis* than the non-antibiotic treatment (3/3 vs. 0/1). When the two species were combined and considered as streptococci, however, the antibiotic-treated cases had a better cure rate than the control group (5/8 vs. 2/6).

For cases caused by *E. coli*, there were no significant differences in BC between the groups that received antibiotic treatment and those that did not in the studies by Keller and Sundrum (80.0% vs. 60.0 and 50.0%), Ganda et al. [24] (80.0% vs. 85.0%), and Suojala et al. [21] (90.5% vs. 86.8%). In Klostermann et al. [22], one case of *E. coli* was cured by antibiotic administration compared with the control group, in which one case of *E. coli* was not cured. The antibiotic-treated cases due to coliform pathogens were cured bacteriologically in 87% of the cases in the study by Morin et al. [23], with no significant difference compared to the control group (72%). The antibiotic treatment performed better for infections caused by *Klebsiella* spp. in the study by Ganda et al. [24], but the difference was not significant.

### 2.6. Risk Difference

**Figure 2:**
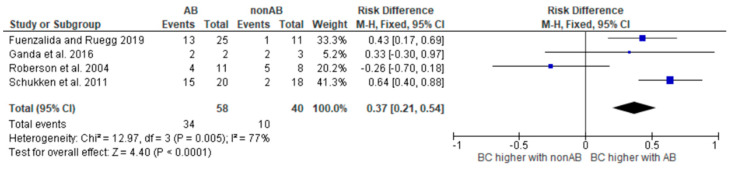
Risk difference diagram for *Klebsiella* [16,17,19,24].

**Figure 3:**
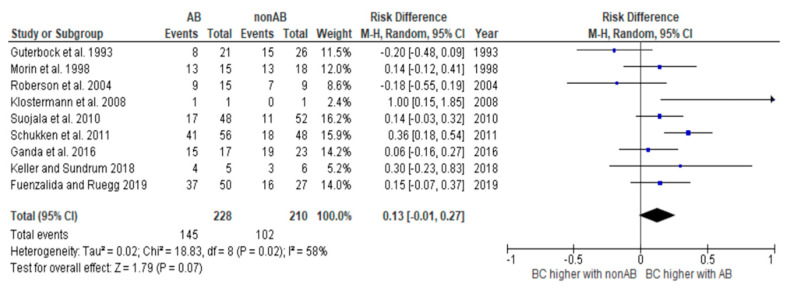
Risk difference diagram for coliforms [16,17,18,19,20,21,22,23,24].

**Figure 4:**
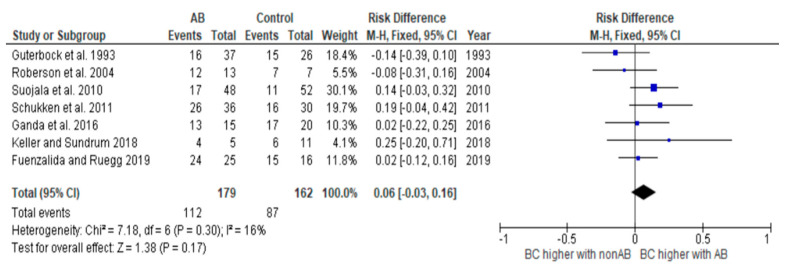
Risk difference diagram for coliforms without *Klebsiella* [16,17,18,19,20,21,24].

**Figure 5:**
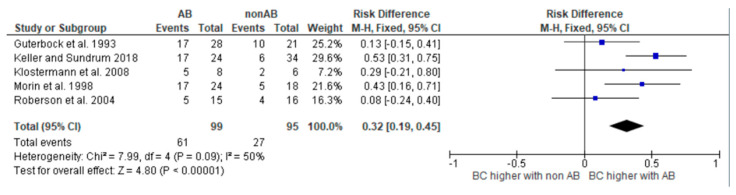
Risk difference diagram for Streptococci [16,19,20,22,23].

To meet the aim of our PICO, which is to determine the difference between the cure rates between the antibiotic-treated and non-antibiotic-treated groups for the different pathogens, we created risk difference diagrams for each pathogen group (Figure 2, Figure 3, Figure 4 and Figure 5). It is known that many factors can influence the cure rate. However, since we only selected studies that were balanced concerning other influencing factors, the difference is still robust.

In two studies, only relative cure rates were stated [17,18]. However, information on the absolute number of cases was required to create the risk difference diagrams. Therefore, for these two studies, the absolute number of cases was back-calculated using the information in the text.

The diagrams show the risk differences in BC when comparing antibiotic-treated cases with non-antibiotic-treated cases. The risk difference represents the difference in the cure rate between the groups. For each study, the graph shows a line indicating the overall 95% confidence interval. The point in the mean value represents the average value. If this point is in the right half, the probability of a cure in this study is higher in the group treated with antibiotics. However, if the point is on the left-hand side, the BC is higher in the group not treated with antibiotics.

The heterogeneity of the study analysis, which contained all studies with coliform microorganisms, could be removed by removing the studies with *Klebsiella*. The analysis of all studies containing *Klebsiella* continued to show significant heterogeneity. These results should therefore be interpreted with caution. Further studies are required.

## 3. Discussion

With the growing resistance of microorganisms to antibiotics, one goal is to provide even more evidence-based treatment in the future. To achieve this, the aim of this study was to draw basic conclusions on which cases of mild and moderate clinical mastitis antibiotic therapy is advisable and in which cases it is not. This information should serve as a basis for developing clinical recommendations for the targeted use of antibiotics.

The original plan to carry out a systematic review was changed over the course of the analysis, as in the end, only four studies remained, which were characterized by considerable heterogeneity. Therefore, it would not have been possible to derive valid conclusions. It was thus decided to deviate from the established PRISMA-based systematic review approach and instead include five additional articles that closely adhered to our criteria, albeit with marginal differences. With this approach, it was still possible to make valid statements to improve the relevance and overall quality of the review and to provide orientation for relevant groups of people. These statements must always be evaluated with caution. However, this is generally the case, as there are apparently very few articles on this topic.

Overall, there was no risk of bias in most of the articles. One exception was the study by Guterbock et al. [16], which had elevated risks of bias in several domains. In every study, the different project groups should ideally be balanced concerning animal characteristics, like lactation number, SCC history, number of infected quarters at the cow level, or other relevant variables, before any interventions or treatments are administered. If the groups are not balanced with respect to these baseline characteristics, it will be difficult to attribute the observed results solely to the treatment.

An important factor that can lead to bias is the type of randomization. The aim of randomization is to minimize the influence of confounding variables and to ensure that the groups being compared are comparable at the start of the study, with the exception of the intervention being studied. Good randomization is achieved through rigorous methods, such as computer-generated randomization. This approach ensures an even distribution of potential confounders, increases the validity of the study results, and is increasingly used in newer studies. However, it should be noted that randomization in the field is often difficult, and therefore, especially in older studies, other randomization methods have often been chosen for reasons of practicability. In the study by Schukken et al. [17], randomization was based on ear tag numbers. The use of ear tag numbers as randomization was found to be a suboptimal method in a review by de Jong et al. [4], and thus resulted in an increased risk of bias. A possible explanation could be that with the assignment of an ear tag number to a certain animal, a certain pre-selection has actually already been made. In contrast to this assessment, we decided that this approach does not carry an increased risk of bias. Ear numbers are assigned randomly in the order in which calves are born. For this reason, it is reasonable to assess this approach as random. In addition to rigorous randomization, however, future research should prioritize blinding and protocol standardization to minimize observer bias. The blinding of personnel involved in outcome assessment is especially important in reducing subjective interpretation and unintentional influence during clinical evaluation or data collection [25,26].

Antibiotic treatment does not always result in a significantly higher BC rate for all cases of mastitis. Moreover, even when this is observed, it is not always suitable. Various criteria play a role in deciding whether treatment is appropriate, and each treating person must assess these independently. The results of the risk difference diagrams should help to make a fact-based treatment decision. This decision involves various criteria that each treating person must evaluate independently. However, the results should aid in making a more fact-based treatment decision. There were five studies presenting results for cases caused by streptococci [16,19,20,22,23]. The use of antibiotics resulted in significantly improved BC rates. Looking at the results of the risk difference diagram for streptococci, it can be seen that antibiotic treatment resulted in a 32% higher BC than control treatment. Streptococci are one of the most prevalent mastitis pathogen groups causing mastitis in dairy cows. According to Schmenger and Krömker (2020) [6], 20.2% of mastitis cases in Northern Germany were caused by *S. uberis*. In their study, both cases caused by *S. uberis* and *S. dysgalactiae* showed high BC rates of 73.9% and 82.9% after antibiotic therapy. Considering these numbers and the fact that untreated infections can not only have a significant impact on milk production and milk quality but can also cause lasting damage to udder tissue, it is critical to treat infections caused by streptococci as early as possible with antibiotics.

For cases caused by *E. coli*, there was no significant difference in BC between the groups that received antibiotic treatment and those that did not [18,19,20,21,24]. Several other studies have already demonstrated that these cases have a high tendency to self-cure and to self-limit. For this reason, BC is unlikely to be higher due to an antibiotic cure [27]. Self-cure, also known as spontaneous cure or resolution, refers to the natural ability of an organism’s immune system to combat an infection and restore normal health without external intervention. Günther et al. [28] showed that *E. coli* triggers a rapid and strong inflammation in the udder, with the consequence that the udder’s defense mechanisms are activated just as quickly. In many cases, this already leads to an early eradication of the invading pathogens. Considering these aspects and the fact that the effectiveness of antibiotic therapy is measured by the BC that can be achieved beyond the rate of self-cure, antibiotic treatment is often not indicated in such cases. The studies by Schukken et al. [17] and Klostermann et al. [22] were the only ones in which significantly higher rates of BC were found in cases caused by *E. coli* in the antibiotic group than in the untreated control group. However, it should be noted that the number of cases in Klostermann’s study was too small for this result to be particularly meaningful. In the study by Schukken et al. [17], the authors acknowledged that this result was unusual and offered possible explanations for it. One possibility suggested was that farmer bias could have influenced the results, as farmers were aware of which animals were treated and which were not. This could have led to intervention in the untreated group, which would have been counted as not cured. The authors also pointed out that certain study characteristics, such as the extended treatment duration and the inclusion of only mild and moderate cases, could have contributed to the improved cure rates observed in some cases.

Antibiotic reduction can be achieved by more prudent use or, whenever possible, by omission [2]. It has been shown that mild and moderate cases of CM caused by *E. coli* are an area where antibiotics can be omitted. If one follows the numbers of Schmenger and Krömker (2020) [6], who showed in their study that 32.8% of the coliform infections were mild cases and 42.8% were moderate cases, omission is possible in about 75% of the coliform cases. A different situation arises when there is severe coli mastitis. *E. coli* mastitis can progress rapidly and cause significant damage to the mammary gland and surrounding tissues. In addition, in severe cases, *E. coli* mastitis can lead to bacteria entering the bloodstream, causing bacteremia. As this systemic spread of infection can be life-threatening, it is still recommended to treat those cases systemically with antibiotics to save the animal’s life [7,9,29]. However, in such cases, supportive therapy, for example, by infusion or drenching, is particularly crucial.

*Klebsiella* spp. are not as commonly associated with Gram-negative bovine mastitis as *E. coli,* but can still cause mastitis and are considered one of the major environmental pathogen groups responsible for mastitis. The results of BC in three studies showed better cure rates in the antibiotic-treated group than in the untreated control group [17,18,24]. In the study by Roberson and colleagues, the results were not satisfactory in all treatment groups. *Klebsiella* spp. mastitis can lead to acute and severe cases. The infection can progress rapidly, causing significant udder swelling, heat, and pain. According to a study by Krebs et al. [7], bacteremia was found in 5 of 12 mastitis cases caused by *Klebsiella pneumoniae*. Given the virulence and potential for causing severe infections, *Klebsiella* mastitis requires prompt identification and appropriate management.

It became obvious that regular pathogen diagnostics are definitely useful to avoid unnecessary antibiotic treatments. The use of on-farm rapid tests is a smart option for a quick differentiation of the causative pathogen group, making them a useful tool for deciding on the appropriate treatment. The adoption of modern therapy concepts, including the use of on-farm rapid tests, can lead to significant reductions in antibiotic consumption while achieving consistent cure results [30,31,32].

Overall, various data gaps and deficiencies can be identified in our review. The results were presented for certain common mastitis pathogens, including *S. uberis* and *E. coli*. However, for other typical pathogens, such as *S. aureus* or NaS (Non-aureus-staphylococci), no explicit data was available within our specification of antibiotic treatment vs. non-antibiotic or no treatment in mild and moderate CM. Regarding the results of Schmenger and Krömker (2020) [6], about 6.0% of CM cases in Northern Germany were caused by NaS and 3.7% by *S. aureus*. While our study illuminates important insights, the gaps in data emphasize the need for further research to comprehensively understand the implications of different treatment approaches across a wider spectrum of mastitis pathogens.

To accurately determine the effectiveness of antibiotic therapy and identify the cases where it works best, an untreated control group is necessary. Due to ethical concerns, the use of an untreated control group is often omitted. Generally, diseased animals must be treated, and the omission of treatment is avoided for animal welfare reasons. However, in this investigation, only mild and moderate cases of CM were considered, where the animal’s life is usually not at risk. However, even such mild and moderate cases of mastitis can lead to pain for the affected animals. Therefore, a possible gain in knowledge and a possible resulting harm to the animals must be carefully weighed in each case. However, it should be kept in mind that such studies, including an untreated control group, are necessary to provide further knowledge that can make future treatment more effective both in terms of animal welfare aspects and in the fight against antibiotic resistance.

It can further be discussed whether it is appropriate to measure the BC in animals that have not been treated with antibiotics at the same time as in the antibiotic-treated animals. Antibiotic treatment results in an active level of the substance in the animal (blood, milk, etc.) over a period of time. This active level protects the affected animal from new infections during this period. In these animals, the sample to determine the BC should be taken after the withdrawal time. In contrast, in the animals not treated with antibiotics, there is no active level, and thus the animals are not antibiotically protected against new infections. For this reason, it is a consideration to take the sample for the determination of BC earlier in these animals in the future.

For further studies, it is advisable to present outcome variables such as BC not only as percentages but also to always include absolute numbers. In addition, in studies that include all cases of CM, not only should the severity of the mastitis be taken into account but an analysis should also be adapted to the clinical severity should be made, as this plays a decisive role in the treatment decision. A separate evaluation of cases based on different pathogen groups can further improve the specificity of the results.

Standardized clinical trials play a crucial role in advancing scientific knowledge and increasing the information value of research findings. The use of uniform protocols, well-defined outcome measures, and stringent statistical analyses not only improves the quality of evidence but also promotes collaboration and data sharing among researchers. By promoting transparency and reducing bias, standardized clinical trials help establish evidence-based practices, ultimately benefiting affected animals, veterinarians, and policymakers alike.

## 4. Materials and Methods

### Search and Study Selection Process

We conducted this review in accordance with the guidelines of the PRISMA statement [33]. The review protocol has been available at https://www.muhh.eu/forschungsprojekte since November 2022. The search for relevant studies was conducted via Pubmed (https://pubmed.ncbi.nlm.nih.gov/) and Web of Science (https://webofknowledge.com/WOS) on September 28th, 2022. We created a list of search terms containing the PICO elements, including their synonyms or other related words [34]. Accordingly, the search terms included the words “cows”, “mastitis”, “antimicrobials”, and synonyms. To ensure the quality of the developed search string, ten relevant studies known to the authors were selected prior to the search. If these pre-selected studies were not represented in the search, minor modifications to the search string were considered. Studies on subclinical mastitis treatment and dry-off treatments were excluded from the investigation. No date restrictions have been applied regarding the publication date.

The literature search results were saved in Excel files. Deduplication was performed by manual check-up. The following selection process of the studies had two steps. During the first step, the reviewers (V.K., S.L., F.N., J.N., A.T., N.W., D.K., Y.Z., I.K., S.M., S.T., P.K., and J.K.) screened the title and abstract of each publication to determine eligibility. Studies were eligible if the abstracts were available in English and the articles described trials on mild and moderate clinical cases of bovine mastitis with natural disease exposure, with at least one intervention group receiving antibiotic therapy during lactation. Records were excluded if one of these criteria did not apply.

The details of articles remaining after the abstract screening were saved in an Excel file and moved on to step two, the full-text screening. For this, the participating reviewers were divided into junior and senior groups, with one member from each group forming a pair. The articles were divided between these pairs, and the entire texts were read by both partners of each pair. It was ensured that the reviewers were assigned different articles than in step one. The Excel file was completed with more detailed information about the articles after the reviewers had compared their results. Any disagreements between the independent reviewers at all stages were resolved through discussion with another reviewer until a consensus was reached. During the full-text screening, articles remained if the full-text was available in the English language, the studies included groups that were limited to non-severe mastitis cases in dairy cattle breeds during the lactation period (or if severe cases were also included, separate results for BC rate for the non-severe cases were available), the study was an RCT with at least one appropriate comparison group receiving a non-antibiotic treatment method, a placebo, or no treatment, and BC was measured at a minimum of 14 days after the onset of therapy. In addition, only studies in which the different treatment groups were balanced concerning animal factors (like lactation number) were included.

The reviewers extracted relevant data from the remaining studies, including author and year of publication, period of data collection, geographical location (country), number of farms, number of groups, number of units analyzed (n), level of intervention (quarter or cow), inclusion criteria/cow eligibility (e.g., cow-factors), definition of severity, case identification, antibiotic(s) and non-antibiotic substances used, dose of antibiotics and non-antibiotics used, route of administration, frequency of administration, and treatment duration. Study groups that received a combination of a non-antibiotic treatment and an antibiotic treatment were not included. The outcome of interest was BC at a minimum of 14 days after treatment onset in each of the respective intervention groups. For this purpose, the following additional data were extracted from the articles: time of measurement (specify the day after administering the intervention), method for identification of mastitis agents, and grouping of identified microorganisms. “Others” and “Mixed” groups were not considered, as they contain very different groups of bacteria, and conclusions on BC would not be relevant here. Unusual pathogens for which results were also only reported in individual studies (e.g., Enterobacter cloacae in Schukken et al. [17]) were also excluded.

V.K., S.L., A.T., J.N., and F.N. assessed the risk of bias for each article using the Cochrane Collaboration’s tool for randomized trials (RoB2 [35]). The reviewers divided the articles among themselves, with each reviewer editing one. The results were then presented and discussed among the group. For each article, the following five areas were evaluated: (1) bias arising from the randomization process, (2) bias due to deviations from intended interventions, (3) bias due to missing outcome data, (4) bias in measurement of the outcome, and (5) bias in selection of the reported results. The result of the algorithm was always considered first. Then, it was discussed whether everyone agreed with this result or not (“assessor’s judgement”).

The meta-analysis was performed using the program RevMan (Review Manager [RevMan], [Computer program], Version 5.4., The Cochrane Collaboration, 2020). We calculated the Mantel–Haenszel summary risk difference. The variation across the studies (heterogeneity) is also given. Sensitivity analyses were performed where heterogeneity was demonstrated.

## 5. Conclusions

The findings revealed that antibiotic treatment resulted in improved bacteriological cure (BC) rates for cases caused by streptococci, highlighting the importance and justification of antibiotic treatment for Gram-positive pathogens like *S. uberis* and *S. dysgalactiae*. However, for cases caused by *E. coli*, there was no significant difference in BC between antibiotic-treated and untreated groups, suggesting that antibiotic therapy may often be unnecessary for mild and moderate coliform infections due to the high tendency of self-cure in these cases and can therefore be avoided. However, for cases caused by *Klebsiella* spp., there is some evidence that antibiotic treatment may be beneficial with regard to BC, so antibiotic therapy may be justified in these cases. Due to the heterogeneity of the analysis, further studies are required.

The study emphasized the importance of regular pathogen diagnostics, like the use of on-farm rapid tests, to avoid unnecessary antibiotic treatments. Implementing modern therapy concepts, including on-farm rapid tests, can lead to significant reductions in antibiotic consumption while achieving consistent cure results, benefiting both animal welfare and the fight against antibiotic resistance.

To enhance future research, presenting outcome variables with absolute numbers, considering severity in analyses, and conducting separate evaluations based on pathogen groups are recommended. The importance of standardized clinical trials with uniform protocols, well-defined measures, and stringent statistical analyses was emphasized to advance scientific knowledge and establish evidence-based practices. In conclusion, this study contributes valuable insights into evidence-based treatment options for mild and moderate CM and emphasizes the need for further research, standardization, and ethical considerations to optimize antibiotic therapy and ensure better animal welfare outcomes while combating antibiotic resistance.

## Figures and Tables

**Table 1:** Study characteristics.

Study	Demographics	Method for Identification of Mastitis Agents	BC ^1^: Time of Measurement	Further Evaluated Outcomes
**Guterbock et al., 1993** [16]	3 California dairy herds Total: n = 254 quarters ABT ^2^-a ^3^: n ^4^ = 74 ABT-b ^5^: n = 75 NABT ^6^ n = 105	Cult ^7^	Day 5 (milking 9, ABT a) or day 6 (milking 11, ABT b) and day 21 after initial treatment	CC ^8^
**Roberson et al., 2004** [19]	1 university dairy herd Total: n = 82 cow mastitis events ABT-a: n = 21 ABT-b: n = 19 NABT (c): n = 20 NC ^9^ (d): n = 22	Cult	Days 7 and 36	CC, milk production, disease progression, CMT ^10^ scores
**Schukken et al., 2011** [17]	5 dairy farms in New York State Total: n = 104 cases ABT: n = 56 NC= n = 48	Cult & Mol ^11^ (at enrollment: identification of coliform pathogens mostly via on-farm culture; confirmation in laboratory)	Days 7 ± 2 and 14 ± 2	Clinical improvement, milk production, linear score, survival probability
**Fuenzalida and Ruegg, 2019** [18]	2 commercial Wisconsin dairy herds Total: n = 168 cases ABT-a: n = 56 ABT-b: n = 56 NC: n = 56	Cult (at enrollment: identification of Gram-negative pathogens via on-farm culture; confirmation in laboratory)	Days 14 and 21	Quarter-level CM ^12^ recurrence, cc, culling within 21 days after enrollment, voluntary dry-off of an affected quarter, days until cc, days of milk discard, days to BC, days to culling, voluntary quarter dry-off, daily milk production

^1^ bacteriological cure, ^2^ antibiotic treatment, ^3^ antibiotic treatment: treatment a, ^4^ number, ^5^ antibiotic treatment: treatment b, ^6^ non-antibiotic treatment, ^7^ cultural, ^8^ clinical cure, ^9^ negative control, ^10^ California Mastitis Test, ^11^ molecular, ^12^ clinical mastitis.

**Table 2:** Interventions and comparators.

Study	Intervention	Comparator
Guterbock et al., 1993 [16]	ABT ^1^-a ^2^: IMM ^3^ amoxicillin (62.5 mg, every 12 h, 3 times ABT-b ^6^: IMM cephapirin (200 mg, every 12 h, 2 times)	Oxytocin IM ^4^ (100IU ^5^, every 12 h, 2 or 3 milkings) (=NABT ^7^)
Roberson et al., 2004 [19]	IMM amoxicillin (62.5 mg, every 12 h, 3 times)	Frequent milk-out (+20IU Oxytocin) (=NABT) No treatment (=NC ^8^)
Schukken et al., 2011 [17]	IMM ceftiofur hydrochloride (125 mg in 10 mL, 5 days, every 24 h)	No treatment (=NC)
Fuenzalida and Ruegg, 2019 [18]	ABT-a: IMM ceftiofur hydrochloride (125 mg, 2 days, every 24 h) ABT-b: IMM ceftiofur hydrochloride (125 mg, 8 days, every 24 h)	No treatment (=NC)

^1^ antibiotic treatment, ^2^ antibiotic treatment: treatment a, ^3^ intramammary, ^4^ intramuscular, ^5^ international units, ^6^ antibiotic treatment: treatment b, ^7^ non-antibiotic treatment, ^8^ negative control.

**Table 3:** Results of the risk of bias analysis.

Study	Domain 1	Domain 2	Domain 3	Domain 4	Domain 5	Total
**Guterbock et al. (1993)** [16]	!	+	+	!	+	!
**Roberson et al. (2004)** [19]	+	+	+	+	+	+
**Schukken et al. (2011)** [17]	+	+	+	+	+	+
**Fuenzalida and Ruegg (2019)** [18]	+	+	+	+	+	+

+ (low), ! (some concerns).

**Table 4:** Bacteriological cure results by pathogen from the initial four articles.

Bacterial Group/Species	BC ^1^ Results ABT ^2^-a ^3^ (n ^4^/n, %)	BC Results ABT-b ^5^ (n/n, %)	Total Results of ABT	BC Results Comparator NC ^6^ (n/n, %)	BC Results Comparator NABT ^7^ (n/n, %)	Total Results of Comparators	Study
Streptococci	6/13 (46.2)	11/15 (73.3)	17/28 (60.7)		10/21 (47.6)	10/21 (47.6)	Guterbock et al., 1993 [16]
	3/4 (75)		3/4 (75)	2/7 (29)	2/9 (22)	4/16 (25)	Roberson et al., 2004 [19]
*E. coli*	(100.0)	(92.9)		(93.7)			Fuenzalida and Ruegg, 2019 [18]
	(89.0)			(53.0)			Schukken et al., 2011 [17]
	8/9 (89)		8/9 (89)	4/4 (100)	3/3 (100)	7/7 (100)	Roberson et al., 2004 [19]
Coliforms	8/21 (38.1)	8/16 (50.0)	16/37 (43.2)		15/26 (57.7)	15/26 (57.7)	Guterbock et al., 1993 [16]
*Klebsiella pneumoniae*	(42.9)	(63.6)		(9.1)			Fuenzalida and Ruegg, 2019 [18]
*Klebsiella* spp.	(57.0)			(19.0)			Schukken et al., 2011 [17]
	2/7 (29)		2/7 (29)	3/5 (60)	2/3 (67)	5/8 (62.5)	Roberson et al., 2004 [19]

^1^ bacteriological cure, ^2^ antibiotic treatment, ^3^ antibiotic treatment: treatment a, ^4^ number, ^5^ antibiotic treatment: treatment b, ^6^ negative control, ^7^ non-antibiotic treatment.

**Table 5:** Study characteristics of the additional five articles.

Study	Demographics	BC ^1^: Time of Measurement	Intervention	Comparator	Deviation from the Initial Criteria
Morin et al., 1998 [23]	1 university dairy herd in Illinois Total: 172 clinical mastitis cases in 124 cows ABT ^2^: 90 NC ^3^: 82	Day 14	SevSc ^4^ 1: 200 mg of cephapirin sodium IMM ^5^ SevSc 2: like SC1, additional oxytetracycline (16.5 mg/kg IV ^6^, q 24 h) SecSc 3: oxytetracycline (16.5 mg/kg IV 24 h) All SecSc´s: additional supportive treatment as the respective control group	SevSc 1: complete milk out by administering oxytocin (20U, IV or IM ^7^) (=NABT ^8^**)** SevSc 2: like SC 1, with an additional stripping at 12 pm SevSc 3: stripping every 3 h, additional flunixine meglumine (1.1 mg/kg q 8 h) and fluids (in case of dehydration)	Proportion of severe cases: Antibiotic treatment group: 19 (21%) Control group: 20 (24%)
Klostermann et al., 2008 [22]	4 adjacent herds in Ireland Total: n = 50 quarters in 48 cows ABT: 25 quarters NABT: 25 quarters	Day 14	150 mg penethamate hydriodide, 150 mg dihydrostreptomycin (as sulphate), 50 mg framycetin sulphate and 5 mg prednisolone; infused at 24-h intervals on days 1, 2 and 3	lacticin 3147, on days 1, 2 and 3 with 24-h intervals (=NABT)	Proportion of severe cases: Antibiotic treatment group: 10/18 Control group: 10/17
Suojala et al., 2010 [21]	61 typical Finnish dairy herds Total: 132 cows ABT: 64 cows NC: 68 cows	Days 2 and 21	enroflocaxin (5 mg/kg), 2 d, every 24 h, first dose IV, second dose SC ^9^; additional ketoprofen IV or IM (3 mg/kg) or 4 mg/kg per os daily for 1 to 3 days	Ketoprofen (3 mg/kg IM or 4 mg/kg per os daily for 1 to 3 days) (=NABT)	Proportion of severe cases: 80.1% (n = 105) of the cows had moderate to severe signs 19.9% (n = 26) had mild signs
Ganda et al., 2016 [24]	1 dairy herd in New York Total: 80 cows ABT: 40 cows NC: 40 cows	Days 10 and 14	ceftiofur (125 mg) IMM, 5 days	No treatment (=NC)	it is not apparent how many cases were mild, moderate, or severe, and this was not included in the statistical analysis
Keller and Sundrum, 2017 [20]	4 German dairy herds (1 organic, 3 conventional) Total: n = 180 dairy cows n = 60 for each treatment group	Days 7, 14 and 28	Veterinarian’s decision: Synulox LC Plus, Cloxamycin L, Oxacillin-Na 1000 mg-Euter-Injektor, Vetriclox L, Peracef, Ubrolexin, Procain-Penicillin-G Injector or Wedeclox Mastitis	homeopathic treatment (21 remedies, 10 globules per day, 5 days) placebo treatment (Globuli Sacchari HAB Gr. 3, 10 globules per day, 5 days)	Determining BC at day 7

^1^ bacteriological cure, ^2^ antibiotic treatment, ^3^ negative control, ^4^ severity score, ^5^ intramammary, ^6^ intravenous, ^7^ intramuscular, ^8^ non-antibiotic treatment, ^9^ subcutaneous.

**Table 6:** Bacteriological cure results by pathogen, supplemented with the results from the five additional articles.

Bacterial Group/Species	BC ^1^ Results ABT ^2^-a ^3^ (n/n, %)	BC Results ABT-b ^4^ (n/n, %)	Total Results of ABT	BC Results Comparator NC ^5^ (n/n, %)	BC Results Comparator NABT ^6^ (n/n, %)	Total Results of Comparators	Study
***S. uberis***	11/17 (64.7)		11/17 (64.7)	3/16 (18.7)	1/12 (8.3)	4/28 (14.3)	Keller and Sundrum, 2017 [20]
	3/3 (100.0)		3/3 (100.0)		0/1 (0.0)	0/1 (0.0)	Klostermann et al., 2008 [22]
***S. dysgalactiae***	6/7 (85.7)		6/7 (85.7)	2/3 (66.7)	0/3 (0.0)	2/6 (33.3)	Keller and Sundrum, 2017 [20]
	2/5 (40.0)		2/5 (40.0)		2/5 (40.0)	2/5 (40.0)	Klostermann et al., 2008 [22]
**Streptococci**	6/13 (46.2)	11/15 (73.3)	17/28 (60.7)		10/21 (47.6)	10/21 (47.6)	Guterbock et al., 1993 [16]
	3/4 (75)		3/4 (75)	2/7 (29)	2/9 (22)	4/16 (25)	Roberson et al., 2004 [19]
	17/24 (71)		17/24 (71)	5/18 (28)		5/18 (28)	Morin et al., 1998 [23]
***E. coli***	4/5 (80.0)		4/5 (80.0)	3/5 (60.0)	3/6 (50.0)	6/11 (54.5)	Keller and Sundrum, 2017 [20]
	(100.0)	(92.9)		(93.7)			Fuenzalida and Ruegg, 2019 [18]
	(89.0)			(53.0)			Schukken et al., 2011 [17]
	8/9 (89)		8/9 (89)	4/4 (100)	3/3 (100)	7/7 (100)	Roberson et al., 2004 [19]
	1/1 (100.0)		1/1 (100.0)		0/1 (0.0)	0/1 (0.0)	Klostermann et al., 2008 [22]
	16/20(80.0)		16/20 (80.0)	17/20 (85.0)		17/20 (85.0)	Ganda et al., 2016 [24]
	38/42 (90.5)		38/42 (90.5)	33/38 (86.8)		33/38 (86.8)	Suojala et al., 2010 [21]
**Coliforms**	8/21 (38.1)	8/16 (50.0)	16/37 (43.2)		15/26 (57.7)	15/26 (57.7)	Guterbock et al., 1993 [16]
	13/15 (87)		13/15 (87)	13/18 (72)		13/18 (72)	Morin et al., 1998 [23]
***Klebsiella pneumoniae***	(42.9)	(63.6)		(9.1)			Fuenzalida and Ruegg, 2019 [18]
***Klebsiella* spp.**	(57.0)			(19.0)			Schukken et al., 2011 [17]
	2/7 (29)		2/7 (29)	3/5 (60)	2/3 (67)	5/8 (62.5)	Roberson et al., 2004 [19]
	2/2 (100.0)		2/2 (100.0)	2/3 (66.7)		2/3 (66.7)	Ganda et al., 2016 [24]

^1^ bacteriological cure, ^2^ antibiotic treatment, ^3^ antibiotic treatment: treatment a, **^4^** antibiotic treatment: treatment b, ^5^ negative control, ^6^ non-antibiotic treatment.

## Data Availability

No new data were created or analyzed in this study. Data sharing is not applicable to this article.
